# Baicalein, Baicalin, and Wogonin: Protective Effects against Ischemia-Induced Neurodegeneration in the Brain and Retina

**DOI:** 10.1155/2021/8377362

**Published:** 2021-06-29

**Authors:** Li Pan, Kin-Sang Cho, Irvin Yi, Chi-Ho To, Dong Feng Chen, Chi-Wai Do

**Affiliations:** ^1^School of Optometry, The Hong Kong Polytechnic University, Hong Kong; ^2^Schepens Eye Research Institute of Massachusetts Eye and Ear, Department of Ophthalmology, Harvard Medical School, Boston, MA, USA; ^3^Centre for Eye and Vision Research, Hong Kong

## Abstract

Ischemia is a common pathological condition present in many neurodegenerative diseases, including ischemic stroke, retinal vascular occlusion, diabetic retinopathy, and glaucoma, threatening the sight and lives of millions of people globally. Ischemia can trigger excessive oxidative stress, inflammation, and vascular dysfunction, leading to the disruption of tissue homeostasis and, ultimately, cell death. Current therapies are very limited and have a narrow time window for effective treatment. Thus, there is an urgent need to develop more effective therapeutic options for ischemia-induced neural injuries. With emerging reports on the pharmacological properties of natural flavonoids, these compounds present potent antioxidative, anti-inflammatory, and antiapoptotic agents for the treatment of ischemic insults. Three major active flavonoids, baicalein, baicalin, and wogonin, have been extracted from *Scutellaria baicalensis* Georgi (*S. baicalensis*); all of which are reported to have low cytotoxicity. They have been demonstrated to exert promising pharmacological capabilities in preventing cell and tissue damage. This review focuses on the therapeutic potentials of these flavonoids against ischemia-induced neurotoxicity and damage in the brain and retina. The bioactivity and bioavailability of baicalein, baicalin, and wogonin are also discussed. It is with hope that the therapeutic potential of these flavonoids can be utilized and developed as natural treatments for ischemia-induced injuries of the central nervous system (CNS).

## 1. Introduction

Ischemia is a common pathological or traumatic condition accompanied by the reduction of blood supply to the major organs, such as the heart, kidney, intestine, brain, and eye [1]. This leads to an insufficient supply of oxygen and nutrients and an accumulation of metabolic wastes, causing organ damage or failure and resulting in death in severe cases [1]. Neurons in the brain are the most sensitive and vulnerable cells to ischemia. Only a short period of ischemia can elicit irreversible damage to brain tissue, leading to paralysis or death [2, 3]. Stroke was defined by the World Health Organization (WHO) in the 1970s as “rapidly developing clinical signs of focal disturbance of cerebral function, lasting more than 24 hours or leading to death with no apparent cause other than that of vascular origin” [4]. Around 87% of stroke cases are ischemic stroke, which is triggered by a lack of blood supply to focal brain areas, leading to subsequent damage and neurodegeneration [5–7]. Stroke is a leading cause of disability and death worldwide [7, 8]. Thrombolytic medication, such as alteplase (t-PA), is the only FDA-approved therapeutic agent for treating acute ischemic stroke within a few hours after its onset [9]. Given the narrow time window of treatment and high risk of complications, such as hemorrhagic transformation, cerebral edema, and other adverse effects [10], the development of novel neuroprotective therapies against ischemia is paramount.

The visual system is comprised of the sensory organ (eyes) and connecting axon fibers to the visual targets of the brain [11]. Light, as a stimulus, is captured by photoreceptors in the retina, initiating a cascade of chemical and electrical events. The signal is then transferred to the visual center of the brain via the ganglion cell axons of the optic nerve [12–14]. The visual centers process and transform these signals into visual images. Retinal ischemia is frequently involved in various forms of retinal neuropathies, such as age-related macular degeneration (AMD), diabetic retinopathy (DR), glaucoma, and central/branch retinal artery/vein occlusion [15–19]. Following ischemic injuries, a series of events are triggered, including oxidative stress, neovascular and apoptotic changes, and, ultimately, the death of retinal neurons and vision loss [5, 20]. The retina is an extension of the brain in terms of anatomical and embryonic development [21, 22]. The retina also displays similarities to the brain regarding its neuronal and immune responses to injury [22]. The latter is possibly contributed by the structural similarity between the blood-retinal barrier (BRB) and blood-brain barrier (BBB), to which the retina sustains an immune privilege site and shares a similar pattern of immune surveillance and immunoregulatory processes [23, 24]. In response to perturbations in the retina and the brain, innate immunity can be rapidly activated through transcriptional and phenotypic alterations of immune glial cells and the release of inflammatory cytokines [25, 26]. However, excessive activation of innate immune reactivity under injury or traumatic stress can promote further activation of adaptive immunity by antigen-presenting cells that attract and guide peripheral immune cells, such as T cells to the injury sites [27–29]. Neuroinflammation has been well documented as a pathological factor in neuronal death in the brain and in retinal disorders. Because of these similarities, the retina has been commonly considered as an easily accessible indicator of brain disorders, such as Alzheimer's disease (AD), Parkinson's disease (PD), and stroke.

**Figure 1:**
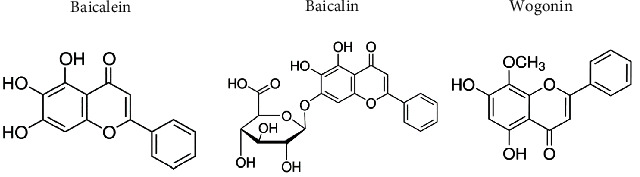
Molecular structures of baicalein, baicalin, and wogonin.

Accumulating evidence suggests that natural herbs exhibit therapeutic potential for the treatment of ischemic stroke [30]. Active ingredients extracted from herbs, including salvianolic acid B and tanshinone from *Salvia miltiorrhiza*, scutellarin from *Scutellaria baicalensis* Georgi (*S. baicalensis*), and honokiol and magnolol from the bark of *Magnolia officinalis*, have been found to have therapeutic potential, because of their antioxidative and anti-inflammatory properties, as well as their ability to maintain BBB permeability [30–36]. The neuroprotective capabilities of other natural extracts, including resveratrol, curcumin, vitamins C and E, and *Gingko biloba*, have also been reported in various CNS disorders [37]. Flavonoids, which are easily accessible by daily consumption of fruits and vegetables, have been found to have high therapeutic efficacy and fewer side effects in both in vitro and in vivo studies [38, 39]. Because of their various pharmacological effects, many flavonoids have demonstrated promising protective effects in the prevention or treatment of various diseases [40–46]. This review mainly focuses on three flavonoids: baicalein (5,6,7-trihydroxyflavone; C_15_H_10_O_5_), baicalin (5,6-dihydroxy-7-O-glucuronide), and wogonin (5,7-dihydroxy-8-methoxy-flavone) (Figure 1), all of which are isolated from the roots of *S. baicalensis*, a widely used herbal medicine in Asian countries [47–51]. Previous studies have demonstrated that baicalein, baicalin, and wogonin have a broad spectrum of biological functions, including antioxidation, anti-inflammation, antiapoptosis, and antiexcitotoxicity [51, 52]. Because of these bioactivities, many published reports have suggested the possibility of developing flavonoids for the treatment of various diseases, including hepatitis, breast cancer, virus infection, and neurodegenerative diseases [43–46]. These pharmacological activities also provide a solid basis for their neuroprotective properties in different models of neuropathies and cognitive impairments. Because of their easy accessibility and low toxicity, baicalein, baicalin, and wogonin may be effective alternatives for the treatment of stroke and other neurodegenerative diseases affecting CNS.

## 2. Bioactivity and Safety of Baicalein, Baicalin, and Wogonin

As the major active flavonoids extracted from *S. baicalensis*, baicalein, baicalin, and wogonin share some common pharmacological properties against inflammation, oxidation, and apoptosis. A comparison of baicalein, baicalin, and wogonin is shown in Table 1, based on the parameters collected from the database of the traditional Chinese medicine lab of systems pharmacology (TCMSP), a database integrating systems biology and pharmacology for drug discovery, development, and understanding of therapeutic mechanisms [53]. The database can be found in the following link (https://old.tcmsp-e.com/tcmsp.php).

Baicalein and wogonin have a lower molecular weight (MW) and a lower value of the topological polar surface area (TPSA) than baicalin, indicating a higher cell membrane permeability of baicalein and wogonin [54]. Furthermore, the permeability of Caco-2 monolayers (intestinal epithelial cells) and the BBB, calculated based on the values of TPSA [55, 56], was found to be higher for baicalein and wogonin, compared to baicalin. In addition, baicalein and wogonin display slower elimination half-time (HL) and higher oral bioavailability (OB) compared to baicalin, suggesting a longer duration of these two flavonoids in systemic circulation. Based on multiparametric guidelines, also known as rules and ligand efficiency (LE) metrics, which determine the extent of druglikeness (DL) [57], all three flavonoids meet the criteria of “drug-like” compounds. This demonstrates the potential of baicalein, baicalin, and wogonin to be easily accessible agents for future clinical use. However, the relatively high hydrophobicity of baicalein and wogonin is reflected by their lipophilicity (AlogP, a logarithm of 1-octanol/water partition coefficient) values [58], compared to that of baicalin. Due to this low water solubility, solvents or carriers may be necessary to enhance the solubility of these flavonoids for therapeutic purposes [50, 59–61].

*S. baicalensis* is a major ingredient in many prescriptions of traditional Chinese medicine (TCM). Numerous studies have been conducted to evaluate its pharmacokinetic profile and bioavailability for its safety and efficacy in clinical applications. It has been reported that baicalein and its metabolite baicalein 6-O-sulfate exist in blood plasma for up to 36 hours after a single oral administration of Xiaochaihu Tang (Sho-Saiko-To) [62], a popular TCM treatment containing extract of *S. baicalensis.* The bioavailability of wogonin, baicalein, and baicalin has also been evaluated in healthy human urine. After a single administration of *S. baicalensis* decoction (equal to 9 g of crude drug), wogonin, baicalein, and baicalin were still detectable in the urine 36 hours postdosing [63]. A similar time profile has also been demonstrated in monkey plasma after three doses of baicalein [64]. The presence of baicalin has been detected in human plasma after administration [65, 66]. Additionally, the safety profile of single or multiple administrations of chewable baicalein tablets has been assessed in healthy subjects. In addition to detecting sustained levels of baicalein and its metabolite baicalin in vivo, these studies revealed that single or multiple doses of baicalein (100–800 mg) were safe and well tolerated with no sign of toxicity in the kidney or liver [67, 68]. These findings implicate the feasibility of developing baicalein, baicalin, and wogonin as safe and long-lasting agents for clinical application.

## 3. Neuroprotective Effects of Baicalein, Baicalin, and Wogonin on the Brain and Retina Ischemia

Neurodegeneration often occurs through the progressive loss of the structure or functions of neurons [69]. Neurodegenerative diseases, such as Parkinson's disease (PD), Alzheimer's disease (AD), and stroke, affect millions of people worldwide, especially in aging populations. The pathogenesis of neurodegeneration is complicated and has been associated with genetics, protein misfolding, intracellular mechanisms, and programmed cell death [70–74]. In ischemic stroke patients, typical symptoms are characterized by the sudden loss of mobility, speaking, or vision unilaterally [75]. Ischemia in the brain can be caused by cardioembolic vessel occlusion, artery to artery embolism, or in-situ small-vessel disease [75]. In the retina, ischemia can result from occlusion of the central or branch retinal vessels or DR [76]. Retinal ischemia may lead to visual impairment and blindness [19].

**Figure 2:**
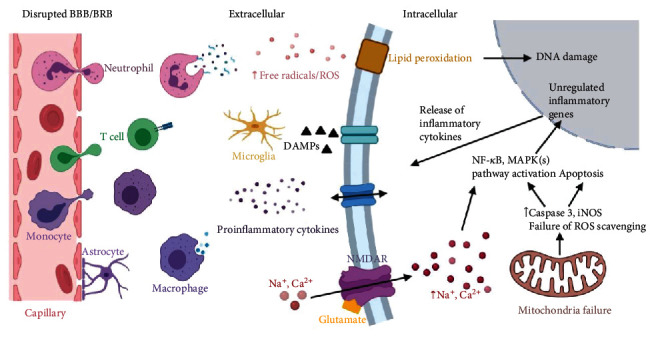
Schematic representation of the main pathological events subsequent to brain ischemia. Created with http://BioRender.com.

Ischemia induces a cascade of neuropathological activities, including oxygen and energy depletion, disruption of ion homoeostasis, glutamate and free radical release, Ca^2+^ channel dysfunction, BBB or BRB disruption, and changes to the inflammatory microenvironment, ultimately leading to cell death and irreversible functional loss [5, 77, 78]. The cellular changes after ischemic stroke are illustrated in Figure 2. There are three major types of stress. First, oxidative stress is induced by rapidly increased reactive oxygen species (ROS) postischemia [79]. This stress can subsequently lead to the peroxidation of membrane lipid, mitochondrial dysfunction, and DNA damage, eventually causing apoptosis and irreversible neuron loss [80, 81]. Second, neuroinflammatory stress, initiated by the activation of innate and adaptive immunity, is a well-known pathological factor in CNS disorders [82, 83]. The resident immune cells (microglia and astrocytes) can be rapidly activated upon sensing damage-associated molecular patterns (DAMPs) released by apoptotic cells [83, 84]. Subsequently, adaptive immune cells can be recruited even with minimized disturbances or disruptions of BBB or BRB postischemia insults, ultimately causing neuron death [29, 69, 85]. Lastly, stress of energy deprivation triggers cytotoxicity at the lesion site. Apart from the excessive free radicals, the disruption of ion homeostasis postischemia/reperfusion injury can lead to overload of Na^+^ and Ca^2+^, which aggravates mitochondrial dysfunction and initiates apoptosis and inflammatory cascades [86]. Furthermore, the release and accumulation of glutamate can cause excitotoxicity of neurons and ultimately leads to cell death [87, 88]. Based on the understanding of neurochemical events under ischemic stress, many neuroprotective therapies are focused on targeting the upstream pathways to reduce damage to the neurons induced by downstream cascades. The protective effects of natural herbs have been demonstrated by many research groups [89]. In the following discussion, the neuroprotective effects of baicalein, baicalin, and wogonin, the major bioactive molecules in *S. baicalensis*, are described based on the sites (i.e., the brain and retina) of postischemic injury.

### 3.1. Ischemia in the Brain

Following ischemic injury in the brain, a cascade of changes is elicited, including oxygen and energy deprivation, increased expression of free radicals and inflammatory cytokines, ion overload, and activation of immune responses [90, 91]. The upstream triggers of these changes and maintenance of tissue homeostasis are recognized as potential therapeutic targets for ischemic stroke treatment. Multiple pharmacological properties of flavonoids as reported in the literature include antioxidant, anti-inflammation, antiapoptosis, and antiexcitotoxicity characteristics that are neuroprotective [92, 93]. The neuroprotective effects of baicalein, baicalin, and wogonin on both in vitro and in vivo ischemic models are discussed (also see Tables 2 and 3 for details).

#### 3.1.1. In Vitro Studies

Free radicals and ROS are known as inducers of cell death postischemic injury. Baicalein has been shown to exert neuroprotective effects and attenuate apoptosis by acting as a scavenger of ROS and nitric oxide (NO) [94]. These baicalein-induced protective effects have been found to be mediated by upregulating the phosphatase and tensin homolog gene (PTEN) and the phosphoinositide 3-kinase (PI3K/AKT) pathway in primary cultures of cortical neurons after oxygen and glucose deprivation (OGD) treatment [94]. Additionally, it has been reported that baicalein improves the survival of HT22 cells after iodoacetic acid- (IAA-) induced oxidative stress, by inhibiting 12/15-lipoxygenase (12/15-LOX) [93], an enzyme which oxidizes polyunsaturated fatty acids to generate bioactive lipid metabolites [95] and is toxic to neurons in neurological disorders [96].

Neuroprotective abilities of wogonin and baicalin have also been reported. In OGD-induced toxicity to rat hippocampal slice culture, pretreatment and posttreatment of hippocampal slices with 50 *μ*M baicalin could significantly prevent cell death, especially in the pyramidal cell layer [97]. Wogonin has been demonstrated to act as an antioxidant and 12/15-LOX inhibitor to improve the survival of primary cortical neurons after H_2_O_2_ or xanthine/xanthine oxidase challenge [98].

It is worth noting that not only oxidative stress but also the dysregulated immune microenvironment can lead to the degeneration of brain cells. In OGD-induced ischemia of PC12 cells, which were derived from a pheochromocytoma of the rat adrenal medulla, baicalein, baicalin, and wogonin all exhibited significant neuroprotective effects by inhibiting oxidation and suppressing inflammation [99]. These protective effects were shown to be mediated by the reduced expression of Toll-like receptor 2 (TLR2), tumor necrosis factor alpha (TNF-*α*), and caspase-3 [99]. Baicalin acts through suppressing OGD-induced phosphorylation of calmodulin- (CaM-) dependent protein kinase II (CaMKII) in primary hippocampal neurons and SH-SY5Y cells [100]. The antiapoptotic effects of baicalin have also been shown to suppress caspase-3 and Bax expression and to promote antiapoptotic factor Bcl-2 expression in hippocampal neurons [100]. The effect of baicalin on rescuing neurons from OGD was comparable with that of CaMKII siRNA knockdown in SH-SY5Y cells, suggesting that baicalin may function as a potent CaMKII inhibitor in neuroprotection [100].

#### 3.1.2. In Vivo Studies

Neuroprotective effects of baicalein, baicalin, and wogonin against ischemic injuries have been reported in various animal models. In the global cerebral ischemia/reperfusion (GCI/R) rat model, Cheng et al. reported that oral administration of 100 mg/kg baicalin for 7 d consecutively can rescue the spatial learning and memory abilities of gerbil significantly, as assessed by the water maze test [101]. Subsequently, Wang et al. showed that baicalin, given at the same dose by intraperitoneal injection for one week immediately after GCI/R injury, can improve the learning and memory abilities in gerbil [100]. These neuroprotective effects exerted by baicalin have been found to be related to the inhibition of CaMKII-mediated downstream biochemical events [100]. CaMKII is an important protein involved in Ca^2+^/glutamate-mediated excitotoxicity under the stress of ischemia [87, 102]. These findings imply that neuroprotective effects arising from baicalin are possibly related to its antiexcitotoxicity capacity. In addition to the CaMKII pathway, Dai et al. found that neuroprotective effects of baicalin can be achieved by its mediation of heat shock protein 70 (HSP70) and mitogen-activated protein kinase (MAPKs) cascades [103]. HSP70 is a critical cytoprotective factor, which is responsible for proper protein folding [104]. And MAPKs are important pathways regulating cell survival and death, including the subgroups of phosphorylated extracellular signal-regulated kinase (pho-ERK), phosphorylated c-Jun N-terminal kinase (pho-JNK), and phosphorylated p38 (pho-p38) [105]. Upregulation of HSP70 and mediation of MAPK subgroups by intraperitoneal administration of baicalin could effectively rescue neurons in the hippocampus after GCI/R injuries [103]. In addition, other studies have shown that baicalin can inhibit the activation of TLR signaling and the relevant downstream inflammatory pathway (i.e., nuclear factor kappa-light-chain-enhancer of activated B cells (NF-*κ*B) pathway) [106, 107]. Following middle cerebral artery occlusion (MCAO), intravenous or intraperitoneal administration of baicalin has been shown to effectively attenuate cerebral infarction by regulating inflammation, including the expression of proinflammatory cytokines TNF-*α* and interleukin-1*β* (IL-1*β*), via TLR2/4 and NF-*κ*B pathway signaling cascades [106, 107]. Baicalin's antioxidative and anti-inflammatory properties are also indicated by decreased levels of both mRNA and iNOS and COX2 protein levels [106, 108].

Similar neuroprotective and antineuroinflammatory effects have been reported with baicalein. For instance, baicalein was found to ameliorate the neurobehavioral deficits and infarct volume caused by small clot embolic stokes (SCEM) or MCAO [93, 94]. Modulating microglia/macrophage M1/M2 polarization and suppressing the NF-*κ*B signaling are suggested to be responsible for the antineuroinflammation and neuroprotection [109]. As a potent antioxidant inhibitor of 12/15-LOX, baicalein was shown to effectively reduce infarct size, edema formation, and 12/15-LOX-induced neuron death in various brain ischemia animal models [92, 110–112]. The inhibitory effect of baicalein is comparable to the protective effects observed in 12/15-LOX knockout mice ALOX15^−/−^ [92]. Pallast et al. found that the protective effects of baicalein are mediated by the suppression of apoptosis-inducing factor (AIF) nuclear translocation in an MCAO model [113]. Later, in addition to confirming the antiapoptotic effect through AIF regulation, Li et al. reported the involvement of the poly (ADP-ribose) polymerase-1 (PARP-1)/AIF pathway in baicalein-induced neuroprotection [114]. Furthermore, inflammation-related factors, such as cytosolic phospholipase A2 (cPLA2) and p38 MAPK, have been reported to be downregulated after baicalein administration in the MCAO rat model [112, 115]. The alteration of peroxisome proliferator-activated receptor *γ* (PPAR*γ*) and nuclear translocation induced by brain ischemia/reperfusion injury have also been reported to be returned to the balanced stage by baicalein pretreatment [116].

Wogonin is also found to exert neuroprotective effects on both GCI/R and MCAO rat models. In the GCI/R rat model, wogonin was shown to effectively attenuate neuron loss and histological changes in the hippocampal CA1 region [117]. The expression of inflammatory mediators (e.g., iNOS and TNF-*α*) at the injury site was significantly suppressed by intraperitoneal administration of wogonin in rats after ischemia injury [117]. In the MCAO rat model, wogonin pre- and posttreatment was shown to both alleviate the infarct volume and behavioral deficits and promote angiogenesis in the peri-ischemia area [118, 119]. Upregulation of transforming growth factor beta (TGF-*β*1) expression in the ischemic brain tissue was observed in wogonin-treated rats 2 weeks after ischemic injury [119]. TGF-*β*1 has previously been reported as an important regulator of angiogenesis in hypoxic tissue [120]. This finding indicates that wogonin protects neurons by promoting microvascular formation and subsequently restoring blood supply via the TGF-*β*1 pathway. The vasodilatory effect of wogonin mediated by inhibition of both intracellular Ca^2+^ release and extracellular Ca^2+^ influx could be a potential treatment paradigm for ischemia [121]. This evidence strongly supports the feasibility of developing baicalein, baicalein, and wogonin as candidates for neuroprotection following ischemic stroke.

### 3.2. In Retina Ischemia

**Figure 3:**
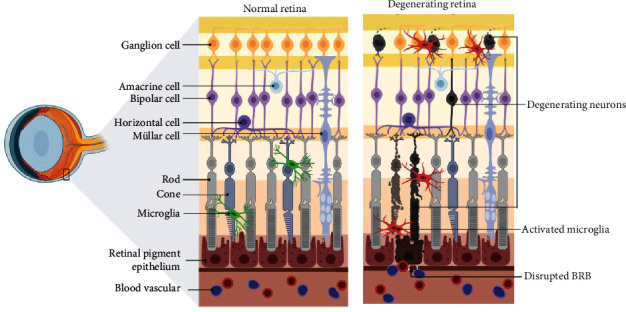
Schematic diagram showing the normal retina and degenerating retina resulting from ischemia. Created with http://BioRender.com.

Injuries caused by retinal ischemia are common in many ocular disorders, such as central/branch retinal artery/vein occlusion, DR, glaucoma, and AMD [15–19]. Similar to ischemic brain injuries, retinal ischemia triggers oxidative stress, inflammation, neovascularization, and, ultimately, the death of retinal neurons [5, 20].  Figure 3 illustrates the morphological changes to the retina and neuron death after ischemia-induced stress. In the following, the antioxidative, anti-inflammatory, antiapoptotic effects of baicalein, baicalin, and wogonin after retinal ischemia for in vitro and in vivo studies are summarized (Tables 4 and 5).

#### 3.2.1. In Vitro Studies

The antioxidative properties of baicalein against retinal ischemia were first reported by Liu et al. [122]. Baicalein was shown to significantly increase cell viability of human retinal pigment epithelium cells (hRPEs) against H_2_O_2_-induced oxidative stress by scavenging ROS and suppressing the production of matrix metalloproteinase-9 (MMP-9) and vascular endothelial growth factor (VEGF) [122]. Subsequently, the antioxidative effects of baicalein on dissociated primary rat retinal cells were demonstrated to be subjected to ascorbate- and FeSO_4_-induced oxidative stress [123]. Baicalein pretreatment not only suppresses the expression of hypoxia-inducible factor-1*α* (HIF-1*α*), VEGF, and MMP-9 but also increases the level of heme oxygenase-1 (HO-1) in ascorbate- and FeSO_4_-stimulated retinal cells [123]. These findings indicate that baicalein has strong antioxidative capabilities in retinal cells, which are comparable to the antioxidative effects exerted by Trolox (6-hydroxy-2,5,7,8-tetramethylchroman-2-carboxylic acid) [123]. In addition, baicalein and wogonin have been found to demonstrate anti-inflammatory effects by suppressing *IL-6* and *IL-8* expression in IL-1*β*-challenged ARPE-19 cells, while NF-*κ*B binding activity is suppressed by wogonin [124]. Similar anti-inflammatory effects have been reported by other recent studies. Chen et al. found that wogonin effectively suppressed LPS-induced activation of the TLR4/NF-*κ*B pathway and subsequently increased expression of inflammatory cytokines IL-1*β*, IL-6, IL-8, cyclooxygenase-2 (COX-2), TNF-*α*, and iNOS in ARPE-19 cells [125]. It was also reported that administration of wogonin to endoplasmic reticulum- (ER-) challenged ARPE-19 cells increased the expression of tight junction proteins claudin-1 and ZO-1 [126]. In another study which mimicked DR in vitro by treating ARPE-19 cells and human retinal microvascular endothelial cells (HRMECs) with high glucose, baicalin was shown to exert antiapoptotic effects by inhibiting the release of proinflammatory cytokines and ROS [127]. These protective effects of baicalin are likely mediated by the suppression of NF-*κ*B and p38 MAPK pathways by upregulating miRNA-145 expression [127].

#### 3.2.2. In Vivo Studies

In the rat retinal ischemia/reperfusion model, baicalein pretreatment effectively regulated the expression of apoptotic factors, including Bax and Bcl-2, subsequently reducing retinal cell apoptosis [123]. In addition, baicalein pretreatment significantly improved the inner retinal functions in electroretinogram (ERG) assessments [123]. Similar neuroprotective effects were also observed in baicalin-treated rats with ischemia/reperfusion injury. A reduced loss of Thy-1^+^ neuron cells and reduced expression of apoptosis markers, including caspase-3 and 8 and poly (ADP-ribose) polymerase-1 (PARP-1), have been found in the retina after ischemic insults [128]. In addition, the downregulated expression of GFAP after baicalin treatment indicates the involvement of baicalin in regulating glial responses and neuroinflammation [128], in addition to its antioxidant and antiapoptosis properties.

The anti-inflammatory effects of baicalein and baicalin have been demonstrated in rats with DR. The excessive activation of resident retinal immune cells is widely known as a pathogenic factor of neurodegeneration in retinal disorders [84, 129]. Yang et al. reported the occurrence of vascular abnormality and RGC loss in the DR rat retina in combination with activation of microglia and Müller cell dysfunction [130]. Oral administration of baicalein has been shown to significantly protect retinal vessels and neurons from DR-induced dysfunction and apoptosis through suppressing the activation of retinal inflammatory processes modulated by microglia and Müller cells and by reducing the release of proinflammatory cytokines, including IL-18, TNF-*α*, and IL-1*β* [130]. The protective effects of baicalin are believed to function through inhibiting the expression of apoptosis regulators, including Bax and Bcl-2, on the RGC layer [131]. Intraperitoneal application of baicalin was found to inhibit the otherwise-elevated aldose reductase activity (ARA) in diabetes. This suggests that baicalin acts as an aldose reductase inhibitor, potentially retarding the progression of apoptosis induced by diabetes [131].

In addition to oxidative stress and inflammation, vascular hyperpermeability of retinal blood vessels has been suggested to be a pathogenic factor in retinal ischemia, DR, and AMD. Othman et al. reported that 12/15-LOX activation leads to vascular hyperpermeability in DR by inhibiting protein tyrosine phosphatase and activating VEGF-R2 signaling pathways [132]. As a potent inhibitor of 12/15-LOX, baicalein significantly reduces the lipid metabolites of 12- and 15-hydroxyeicosatetreanoic acids (HETE) expressed during 12/15-LOX activation in DR [132]. The HETE-induced upregulation of NOX2 and ROS is also reported to be downregulated by baicalein treatment in *Ins2^Akita^* diabetic mouse retina. Likewise, baicalein has been shown to protect against HETE-induced vascular hyperpermeability by acting as a VEGF-R2 inhibitor, restoring phosphoserine phosphatase-1 (pSHP1) levels in DR [132]. The inflammatory cytokine IL-6 and intracellular adhesion molecules (ICAMs) ICAM-1 and vascular cell adhesion molecule-1 (VCAM-1) were remarkably inhibited in the diabetic retina [132]. Taken together, baicalein functions as a 12/15-LOX inhibitor, mediating its effects primarily on vascular and retinal barriers.

## 4. Pathways Targeted by Baicalein, Baicalin, and Wogonin during Neuroprotective Processes

**Figure 4:**
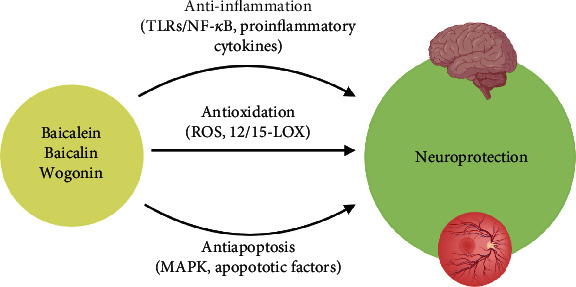
Targeted pathways initiated by baicalein, baicalin, and wogonin demonstrating the neuroprotective effects in the brain and retina. Created with http://BioRender.com.

As described above, the neuroprotective effects triggered by baicalein, baicalin, and wogonin are possibly related to their anti-inflammatory, antioxidative, and antiapoptotic capabilities (Figure 4).

Firstly, the antioxidative effects of baicalein, baicalin, and wogonin are recognized by their ROS scavenging properties. Under normal circumstance, ROS play a pivotal role in many biological processes, such as redox balance in cells. However, a dramatic increase of ROS production may disturb this homeostatic balance under oxidative stress conditions (e.g., ischemia), eventually leading to cell death. Typically, scavenging excessive ROS is a neuroprotective target in neurodegenerative disorders [133]. Baicalein and baicalin serve as potent 12/15-LOX inhibitors with high antioxidative efficiencies. 12/15-LOX is found to be upregulated after stroke, resulting in neuronal death and leakage of BBB and BRB [134]. Inhibition of 12/15-LOX was also shown to reduce infarct volume and edema in the stroke area, suggesting its potential role as an effective and viable therapeutic option for ischemia [92, 135, 136]. Furthermore, the protection of BBB and BRB help preserves the microenvironment at the injured site from disruption by systemic immunity and secondary inflammatory responses caused by degeneration.

Secondly, the anti-inflammatory effect initiated by these flavonoids after ischemia is revealed by the suppression of proinflammatory cytokine release. Excessive activation of microglia has been emergingly reported as a pathogenesis for the development of neurodegenerative diseases [137]. In addition, baicalein and baicalin exhibit the capability in regulating microglia homeostasis after ischemic stress [109]. These properties have been demonstrated to be mediated through interactions with TLR/NF-*κ*B and PARP-1/AIF pathways [99, 106, 107, 124, 125, 127].

Lastly, the antiapoptotic capacity of baicalein, baicalin, and wogonin has been reported in ischemia-injured brain and retina. Through modulating the MAPK pathway and the production of apoptotic factors, these flavonoids effectively rescue neurons in the brain and retina [100, 112, 113, 115, 123, 127, 128, 131]. In addition, the blockage of ion channel dysfunction after ischemic stress remarkably ameliorates the excitotoxicity caused by ion overload and subsequently decreases neuronal death [86, 121]. Taken together, all the evidence strongly supports the feasibility of developing these flavonoids as natural neuroprotectants.

## 5. Conclusion and Future Directions

Ischemia often results in physical disabilities, including paralysis and blindness. This is particularly common in the aging population. Currently, effective clinical intervention for ischemia-induced damage is limited. Ample evidence has demonstrated the potent neuroprotective properties of baicalein, baicalin, and wogonin in both in vitro and in vivo models of ischemia. Flavonoids exert their anti-inflammatory, antioxidative, and antiapoptotic effects on CNS postischemia insults by initiating various potential signaling pathways. Flavonoids are herb extracts, suggesting their potential to be developed as natural neuroprotective agents.

Additional studies are required to elucidate and characterize the pharmacological properties and bioactivities of these flavonoids in combating neuropathies, in order to facilitate the future development of neuroprotectants that are safe and effective. As an extension of the brain, the retina may serve as an easily accessible model and potential therapeutic target site for various neuropathies and other neurodegenerative diseases. Additionally, pharmacodynamic and pharmacokinetic studies, including time- and dose-dependent responses, cytotoxicity, and drug metabolism after systemic and topical administration of these three flavonoids, need to be established. One recent study has reported that the metabolic abilities of flavonoids in the liver and intestines are markedly different among different species, including mice, rats, dogs, monkeys, and humans [138]. In addition, potential targets or receptors through which baicalein, baicalin, and wogonin act on may need to be further characterized and studied. Studies on the combined effects of baicalein, baicalin, and wogonin are limited, raising the possibility that combined flavonoids can achieve longer and stronger protective effects. Lastly, due to the limited water solubility and liposolubility of baicalein, baicalin, and wogonin, formulations and optimizations of these flavonoids, possibly including nanoparticles or other newly developed carriers, may be needed to achieve higher bioactivity and clinical efficacy. It is envisaged that these natural flavonoids can eventually offer new therapeutic therapies for patients with ischemia-induced neural disorders.

## Figures and Tables

**Table 1:** Summary of physical parameters of baicalein, baicalin, and wogonin. MW: molecular weight (Dalton (Da)); TPSA: topological polar surface area (angstroms squared (A^2^)) indicates the membrane permeability; Caco-2: Caco-2 cell monolayer permeability (10^−6^ cm·s^−1^); and BBB: blood-brain barrier permeability (10^−6^ cm·s^−1^) were derived based on TPSA; half-time (HL) (hours (h)), oral bioavailability (OB) (%), the logarithm of 1-octanol/water partition coefficient (AlogP), and drug-likeness (DL) represent the pharmacological properties for each molecule.

Molecule	MW (Da)	TPSA (A^2^)	Caco-2 (10^−6^ cm·s^−1^)	BBB (10^−6^ cm·s^−1^)	HL (h)	OB (%)	AlogP	DL
Baicalein	270.25	90.9	0.63	−0.05	16.25	33.52	2.33	0.21
Baicalin	460.42	187.12	−1.1	−1.97	—	29.53	0.84	0.77
Wogonin	284.28	79.9	0.79	0.04	17.75	30.68	2.56	0.23

**Table 2:** In vitro findings of baicalein, baicalin, and wogonin on different brain cell types.

Cell types	Stimulating molecule(s)	Baicalein		Baicalin		Wogonin		Reference(s)
		Conc.	Effects	Conc.	Effects	Conc.	Effects	
Mouse hippocampal HT22 cell line	20 *μ*M IAA	2 *μ*M	Antioxidant 12/15-lipoxygenase inhibitor					[93]
Primary cortical neurons	Glutamate, OGD, H_2_O_2_, or xanthine/xanthine oxidase	10 *μ*M; 3.5 *μ*M	Promote cell survival through inhibiting 12/15-LOX and removing intracellular ROS and nitrotyrosine reactivities by regulating the PI3/AKT pathway			9.0 *μ*g/ml; 23.7 *μ*g/ml	Improve neuron survival by radical scavenging activity and inhibiting the initiation of LOX-induced apoptosis	[92, 94, 98]
PC-12	OGD	1 *μ*g/ml; 10 *μ*g/ml	Increase survival rate and suppress pro-inflammatory cytokine expression	0.01 mg/ml, 0.1 mg/ml, and 1 mg/ml	Increase survival rate and suppress proinflammatory cytokine expression	0.01 mg/ml, 0.1 mg/ml, and 1 mg/ml	Increase survival rate and suppress proinflammatory cytokine expression	[99]
Primary hippocampal neurons	OGD			1 *μ*M	Protect neurons from apoptosis by suppressing phosphorylation of CaMKII	50 *μ*M	Improve neuron survival on hippocampal slice culture	[97, 100]

**Table 3:** Baicalein, baicalin, and wogonin performance on different brain ischemia models.

Models	Species	Baicalein		Baicalin		Wogonin		Reference(s)
		Conc.	Effects	Conc.	Effects	Conc.	Effects	
SCEM	Rabbit	100 mg/kg	Improve behavioral performance					[93]
MCAO	Rat/mouse	20 mg/kg; 200 mg/kg	Decrease the infarct volume and neurological score; inhibiting 12/15-LOX-induced oxidative toxicity; regulate M1/M2 transformation of microglia/macrophages and block NF-*κ*B nuclear translocation; suppress PARP-1/AIF pathway-induced neuroinflammation; antioxidant 12/15-LOX inhibitor	5, 100, and 200 mg/kg	Reduced the neurological deficit scores, cerebral infarct volume by inhibiting TLR2/4-mediated NF-*κ*B pathway, and expression of iNOS, COX2, and caspase3	20 mg/kg; 50 *μ*M	Reduce infarct area and improve behavior performance through upregulating TGF-*β*1	[92, 94, 106–109, 112–116, 118, 119]
GCI/R	Gerbil/rats			100 mg/kg; 200 mg/kg	Improve learning and memory ability post-I/R injury via anti-inflammatory and antiapoptosis; perform neuroprotection by upregulating HSP70 expression and influencing MAPK cascades in the gerbil hippocampus	0.5, 1, and 10 mg/kg	Increase neuron survival in the hippocampus area by suppressing inflammation (iNOS, TNF-*α*)	[100, 101, 103, 117, 118]

**Table 4:** In vitro findings of baicalein, baicalin, and wogonin on different brain cell types.

Cell types	Baicalein			Baicalin			Wogonin			Reference(s)
	Stimulator	Conc.	Effects	Stimulator	Conc.	Effects	Stimulator	Conc.	Effects	
Primary rat retinal cells	Ascorbate/FeSO_4_	100 *μ*M	Attenuate oxidative stress-induced ROS							[123]
ARPE-19 cells	H_2_O_2_; IL-1b	50 *μ*M; 40 *μ*M	Antioxidation by scavenging ROS; inhibit the expression of MMP-9 and VEGF; suppress IL-6 and IL-8 proinflammatory cytokines	High glucose	50 *μ*M	Protect ARPE-19 cells from apoptosis through upregulating the release of microRNA-145; anti-inflammation by initiating the regulation of NF-*κ*B and p38MAPK signaling pathways	LPS; IL-1 *β*	10 *μ*M or 50 *μ*M; 1–10 *μ*M	Suppress LPS-induced inflammatory responses via the TLR4/NF-*κ*B pathway and protect the tight junctions; inhibit IL-6 and IL-8 via NF-*κ*B pathway suppression; upregulated claudin-1 and ZO-1	[122, 124, 125, 127]
HRMEC	High glucose			High glucose	50 *μ*M	Protect ARPE-19 cells from apoptosis through upregulating the release of microRNA-145; anti-inflammation by initiating the regulation of NF-*κ*B and p38MAPK signaling pathways				[127]

**Table 5:** In vivo findings of baicalein, baicalin, and wogonin on different animal models.

Models	Species	Baicalein		Baicalin		Wogonin		References
		Conc.	Effects	Conc.	Effects	Conc.	Effects	
I/R	Rat	0.5 nmol	Effectively protect retinal cells and electrical functions from oxidation and apoptosis; upregulation of HO-1 and downregulation of HIF-1*α*, VEGF, and MMP-1	12.5 mg/kg	Protect RGCs from retinal ischemia injury and suppress glial cell activity			[123, 128]
DR	Rat; mice	150 mg/kg in rat; 75 mg/kg in mice	Significantly suppressed the inflammatory processes of retinal microglia and Muller cells; enhanced vascular permeability and blood-retina barrier; protect BRB permeability as antioxidant 12/15-LOX inhibitor, anti-inflammation, and antiangiogenesis	150 mg/ml in rat	Protect retinal cells from apoptosis by promoting Bcl-2; perform as an inhibitor of ARA and delay the progression of diabetic retinopathy			[130, 131]

## Data Availability

All data mentioned in this review article are published findings. They have been properly cited in the article.
